# Treatment of Fever by Fresh Air
*The Treatment of Fevers, with Special Reference to Ventilation. By T. Herbert Barker, M.D., F.R.C.S., etc. Read before the Annual Meeting of the British Medical Association at Birmingham, July 30th, 1856. pp. 50. London and Bedford: 1857.


**Published:** 1857-10

**Authors:** 


### TREATMENT OF FEVER BY FRESH AIR.*

Dr. Barker takes a common sense view of what is required in the treatment of fever. He does not deny that medicines are of use; but he—in common with many of the eminent men of the medical profession from the days of Hippocrates to the present time—insists on the necessity of constantly supplying the fever patient with pure air. But the careful nurse fears, perhaps, that the patient will "catch cold" from the draught. Good old lady, you have quite mistaken the characters of your attendant spirits. While you fear to open the window wide to the pure spirit (who will no doubt be a little spiteful if you stint his entrance aperture) you are keeping a very devil in the room, who is able single-handed to defeat all that the doctors are trying to do for the patient's good. But open the window so that the good spirit may chase out the evil one; and you will see what Dr. Barker has witnessed and described.

---

* The Treatment of Fevers, with Special Reference to Ventilation. By T. Herbert Barker, M.D., F.R.C.S., etc. Read before the Annual Meeting of the British Medical Association at Birmingham, July 30th, 1856. pp. 50. London and Bedford: 1857.

"I speak positively in saying that I have never seen anything but good result from these measures; and this sometimes so markedly that the most common-place observer could not but have detected it. If the patient is in the first stage of the disorder, the efficiency of the pure air wards of the second stage; if he is in the second or third stage, the relief is occasionally magical, and can only be compared to what occurs when, during an experiment, an animal is taken out of a chamber containing some narcotic vapour, and is allowed to breathe the pure elements by which life is supported."

